# Occipito-Posterior Presentations

**Published:** 1911-08-26

**Authors:** 


### Obstetrics.

### OCCIPITO-POSTERIOR PRESENTATIONS.

Until quite recently—within the last four or five years—all midwifery text-books have given the frequency of occipito-posterior presentations in figures a great deal smaller than are now believed to represent the facts. The explanation of this apparently egregious error is that in the hospital records from which statistics are taken the patients are not usually seen until late in labour, by which time many presentations originally occipito-posterior have undergone rotation into the occipito-anterior position. Of those which remain unrotated a certain proportion are cases of contracted pelvis or dystocia due to other causes, and hence the teaching has been that the prognosis from occipito-posterior presentations is slightly less good, both for mother and child, than in the normal cases. It is easily seen, however, that if it is true that the proportion of posterior presentations has been underestimated, then the altered view now prevailing entails an acknowledgment that the prognosis of these conditions has been too unfavourably stated; for many of them, hitherto unknown and disregarded, are spontaneously converted to anterior presentations. Accordingly the most modern authorities have modified in their text-books the somewhat too pessimistic statements of the older authors on occipito-posterior presentations.

But above and beyond the modifications in teaching which ensue on the recognition of the larger percentages of occipito-posterior presentations, there is a further tendency noticeable in regard to these cases. Even those cases that are still unrotated fairly late in labour, and even those which are never rotated at all but are delivered with the occiput posterior, are regarded with less disfavour than formerly. An example of this view is to be seen in a paper by Dr. C. B. Ingraham*, which contains an analysis of the course of labour in one hundred consecutive cases of occipito-posterior presentation, All premature deliveries are discarded, as not being fair examples for comparison in difficult labour owing to the smaller size of the children. In fifteen cases there was some contraction of the pelvis, nine times of the generally contracted variety. This figure is slightly greater than that of the mothers admitted taken as a whole, among whom the percentage of deformed pelvis is 13.1. The measurements of the heads of the babies after delivery show an average which is within a decimal point of the normal. It may be assumed, therefore, that the babies were of average size.

In the Johns Hopkins service the percentage of operative deliveries at term is 15.2, a figure which (as in most city hospitals) is somewhat swelled by the admission of patients in whom dystocia has arisen outside the hospital. In this special series of occipito-posterior cases the percentage is 18; and out of these 18, conditions other than the presentation were responsible for the interference 11 times. These conditions included heart disease, contracted pelvis, eclampsia, and so on. The forceps rate strictly attributable to the dystocia of the presentation is therefore only seven per hundred, probably no greater than it would be in occipito-anterior cases. Out of the whole series, the head was delivered with the occiput behind in seven cases; but even of these only one required operative delivery (low forceps). In the other six the second stage of labour averaged but 62 minutes.

The average duration of the labour, both in those with early rotation and those with rotation into the hollow of the sacrum, was practically normal. Perineal tears, too, were 26 per cent., against 23 per cent. for a series of 200 consecutive cases of the anterior variety taken for comparison at the same time. Four tears occurred in the seven patients who were delivered of the occipito-posterior babies.

Of the infants, five died before or within a few days of birth. Three others were somewhat asphyxiated but recovered completely. There were two maternal deaths: one from typhoid fever during which delivery took place, one from chronic myocarditis with dilatation.

Reviewing these figures, the author shows that operative interference need be but little more

* Johns Hopkins Bulletin, January, 1911, p. 501.

frequent than for occipito-anterior presentations. The procedure at the Baltimore clinic, when a posterior presentation is diagnosed, is to leave the condition to Nature. It is objected against those who advocate attempts to bring about rotation artificially that this renders the patient more liable to infection through manipulation. If, however, the head is arrested at the inlet in the occipito-posterior position, version is advised (rather than the high forceps operation or attempts to convert the presentation) as soon as one is convinced that spontaneous advance will not occur; provided of course that the operation is feasible and is not contra-indicated by disproportion between the size of the head and the pelvis.

When the occiput has rotated into the hollow of the sacrum and interference becomes necessary, the forceps is applied in the usual manner to the sides of the head with the pelvic curve towards the face. The occiput is drawn out slowly over the perineum in a horizontal direction until the forehead or root of the nose engages under the symphysis; then the occiput is delivered over the anterior margin of the perineum by slowly elevating the handles.

